# Multicomponent, Tumor-Homing Chitosan Nanoparticles for Cancer Imaging and Therapy

**DOI:** 10.3390/ijms18030594

**Published:** 2017-03-08

**Authors:** Jaehong Key, Kyeongsoon Park

**Affiliations:** 1 Department of Biomedical Engineering, Yonsei University, 1 Yonseidae-gil, Wonju 26493, Gangwon-do, Korea; 2 Department of Systems Biotechnology, Chung-Ang University, Anseong 17546, Gyeonggi-do, Korea

**Keywords:** chitosan, nanoparticles, drug delivery, optical imaging, magnetic resonance imaging, chemotherapy, gene therapy, photothermal therapy, photodynamic therapy, hyperthermic therapy

## Abstract

Current clinical methods for cancer diagnosis and therapy have limitations, although survival periods are increasing as medical technologies develop. In most cancer cases, patient survival is closely related to cancer stage. Late-stage cancer after metastasis is very challenging to cure because current surgical removal of cancer is not precise enough and significantly affects bystander normal tissues. Moreover, the subsequent chemotherapy and radiation therapy affect not only malignant tumors, but also healthy tissues. Nanotechnologies for cancer treatment have the clear objective of solving these issues. Nanoparticles have been developed to more accurately differentiate early-stage malignant tumors and to treat only the tumors while dramatically minimizing side effects. In this review, we focus on recent chitosan-based nanoparticles developed with the goal of accurate cancer imaging and effective treatment. Regarding imaging applications, we review optical and magnetic resonance cancer imaging in particular. Regarding cancer treatments, we review various therapeutic methods that use chitosan-based nanoparticles, including chemo-, gene, photothermal, photodynamic and magnetic therapies.

## 1. Introduction

Cancer is one of the most dangerous human diseases and is the second leading cause of morbidity and mortality in the United States. To date, the incidence of cancer remains constant, but the survival rate has gradually increased from 49%–68% since the 1970s. This can be explained by technological advancements in cancer diagnosis and therapy. Despite enormous investment in cancer prevention and treatment worldwide, cancer statistics indicate the continued need for innovative strategies to eradicate cancer. While patient survival periods have improved, late-stage diagnoses, like metastasis, are still associated with high five-year mortality rates [1]. For most cancers, early detection is closely related to survival rate. For example, the 10-year survival rate for patients with early detected breast, colorectal and prostate cancers is about 80% [2]. Cancer patients with later diagnosis are treated by means of painful and long-lasting chemotherapy and radiation therapy with invasive surgery, with severe side effects, such as hair loss, vomiting, diarrhea and blood disorders.

Cancers are mutated abnormal cells that show uncontrolled proliferation and invasion into neighbor tissues and organs. Currently, the cause of cancer is explained by multiple factors, such as the interplay between genetic mutations and various environmental factors, including foods, viruses, chemicals and ionizing radiation. Nanotechnologies have been applied for cancer therapy due to their ability to overcome the current limitations of cancer diagnosis and therapy.

### 1.1. Nanomedicine for Cancer Treatment

Reported nanoparticles include liposomes, micelles, polymer conjugates, dendrimers, metal and inorganic materials, carbon nanotubes and polymeric nanoparticles. Applications of these materials have been attempted for sustained, controlled or targeted delivery to cancer sites. Furthermore, loading of multiple components into nanostructures has been extensively tested as a strategy to achieve simultaneous cancer diagnosis and therapy, termed theragnosis or theranostics [3].

Owing to their size, nanoparticles can move through microvasculature and across biological barriers. As long as nanoparticles continuously circulate in the body, they have more chances to accumulate in tumor areas, where leaky vasculature and poor lymphatic drainage result, an effect termed enhanced permeability and retention (EPR) [4]. Current nanoparticles basically utilize the passive tumor-homing effect based on EPR.

Particular nanoparticles also may provide various functional groups and several modification options by means of chemical reactions or physical entrapments. For example, hydrophobic segments outside or inside nanoparticles may allow combinations of hydrophobic imaging agents and therapeutic drugs. In particular, many polymeric nanoparticles have been used as diagnostic agents, therapeutic carriers or both [5]. In addition, compared with either free drug or small molecules, such as peptides, polymeric nanoparticles improve the blood half-lives, solubility and stability of imaging probes and chemical drugs while reducing nonspecific accumulation in the body [6].

Among the various types of polymeric nanoparticles, this review will focus on chitosan-based nanoparticles for cancer imaging and therapy. Regarding their imaging purposes, we will review recent applications of chitosan nanoparticles in optical imaging and magnetic resonance (MR) imaging approaches. Regarding their therapeutic applications, we will summarize various therapeutic methods, including chemo-, gene, photothermal, photodynamic and magnetic therapies. Finally, we will summarize the achievements and limitations of current nanoparticles and discuss future prospects.

The first goal of nanomedicine in cancer treatment is to detect early-stage cancer, before metastasis. The survival rates for a number of cancers, such as those of the pancreas, lung and bladder, are significantly related to the stage of cancer at diagnosis. Nanostructures allow incorporation of multiple probes for imaging, which can increase the possibility of detecting early-stage cancer by using multiple imaging modalities. The use of such multicomponent nanoparticles provides diverse information about the body, such as anatomical, physiological and molecular information. The use of multicomponent nanoparticles for multimodal imaging could be promising as a means to overcome the limitations of single imaging modalities with respect to spatial resolution, tissue penetration depth, probe sensitivity, data acquisition time, information provided, cost and clinical relevance (Table 1).

Moreover, the nanoplatform for multimodal imaging makes it possible to visualize tumor margins more clearly, which will be very suitable for minimally-invasive surgical removal of cancer [8]. For example, fluorescent imaging can be applied to fluorescent image-guided surgery in humans. Furthermore, fluorescent-labeled iron oxide nanoparticles enable whole-body MR imaging and fluorescent image-guided delineation of the tumor margin [9,10].

**Figure 1:**
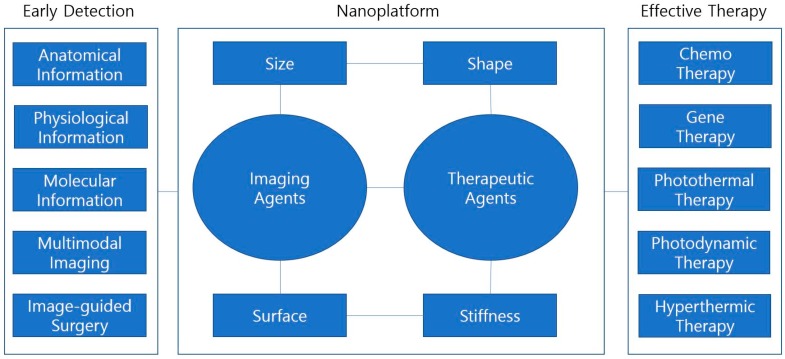
Multicomponent nanoparticles engineered for tumor homing effects.

A nanostructure can also incorporate multiple therapeutic agents and deliver the multiple components to the tumor site. Each therapeutic agent or drug may have its own strengths, and combination therapies may have synergistic effects in cancer treatment. This strategy can minimize the side effects of the therapeutic agents because engineering of nanostructure in terms of size, shape, surface properties and stiffness can significantly increase tumor accumulations [11,12,13,14,15]. Moreover, the ability to load multiple payloads, including imaging and therapeutic agents, into the nanostructure provides both the visualization of nanoparticles at the tumor site and real-time monitoring of the tumor conditions; the resulting information enables the optimization of injection dosage and frequency for cancer therapy and minimizes the side effects (Figure 1).

### 1.2. Characteristics of Chitosan

Chitin is found in natural substances, including the cell walls of fungi and the exoskeletons of arthropods. Chitosan is a deacetylated derivative of chitin and is a linear copolymer consisting of d-glucosamine and *N*-acetyl-d-glucosamine linked by a β-(1→4)-glycosidic bond. It is a positively-charged material depending on the degree of deacetylation of the polymer backbone and can readily bind to nucleic acids, such as DNA and siRNA, by means of electrostatic interactions.

Chitosan has been extensively investigated as an efficient delivery vehicle for various drugs and for genes owing to its excellent biopharmaceutical properties, bioadhesiveness, permeabilization capacity and acceptable toxicity profile [16]. Chitosan is biocompatible and biodegradable owing to its natural origins; thus, it has been widely used as a pharmaceutic adjuvant and presents potential applications as a drug carrier, gene carrier and sensor [17]. Chitosan is subject to enzymatic hydrolysis by both specific enzymes secreted by intestinal microorganisms and non-specific enzymes like lysozymes [18].

The physicochemical properties of chitosan are influenced by its molecular weight, its deacetylation ratio and the presence of any additional moieties. Since the degree of deacetylation is related to the number of protonated amine groups in the chitosan, deacetylation affects solubility and positive charge. For example, acidic conditions below pH 6.5 protonate the amine groups on chitosan, making the chitosan polymer soluble. However, physiological and basic conditions above pH 6.5 prompt deprotonation of the amine groups and precipitation of chitosan [19]. The solubility of chitosan in physiological aqueous solutions is low, representing a major limitation.

Glycol chitosan modified with hydrophilic ethylene glycols is soluble at neutral pH. Hence, glycol chitosan nanoparticles have been developed to deliver therapeutic agents and diagnostic imaging agents [20]. In addition, several chemical modifications of the chitosan chain, such as carboxyl methylation of the hydroxyl group or PEGylation, improve its solubility over broad pH ranges [21,22]. Modified chitosan also enhances the colloidal stability of some hydrophobic materials, such as anti-cancer drugs, allowing its use as a carrier [23,24,25]. Moreover, the pH-sensitive properties of chitosan can be an important feature for effective cancer treatment because free amine groups on the chitosan polymer may act as a “proton sponge” by which chitosan nanoparticles may exit endosomal degradation. Free amine groups attract H^+^ ions from the cytosol, leading to osmotic swelling and rupture of an acidic compartment, like an endosome or lysosome. This mechanism can be very effective in delivering therapeutic agents to cancer cells [26].

## 2. Early Diagnosis of Cancer Using Chitosan Nanoparticles

For the purpose of early cancer detection, in vivo imaging technologies are becoming critical to nanomedicine. With the rapid growth of computing capability, various tomographic whole-body imaging technologies have been designed. Currently, computed tomography (CT) of X-rays, magnetic resonance imaging (MRI) and positron emission tomography (PET) are clinically available. Near-infrared fluorescence (NIRF)-based optical tomography is also usable in small animal preclinical trials. Each of these single imaging modalities provides different information about the body, by means of different imaging acquisition principles. CT scans provide fast and accurate information about anatomical structures that are sensitive to electron-dense elements. MRI scans usually have longer acquisition times than CT scans, but provide differentiable information on soft tissues, representing different relaxation times of hydrogen atoms. PET scans can provide the most sensitive physiological or molecular information about metabolic parameters, such as high-metabolism tumors or infections [7]. While in vivo optical imaging is not utilized on humans owing to its limited tissue penetration depth, it can be combined with another imaging modality, such as MRI or CT in fluorescence-guided surgery approaches that can improve the precision of tumor removal [8]. As emphasized above, the use of a nanostructure allows the incorporation of multiple imaging probes and/or therapeutic agents. More importantly, using nanoparticles of the proper composition can greatly improve the strength of a given imaging modality. In this section, we review MRI, optical imaging and MR-optical multimodal imaging using chitosan nanoparticles.

### 2.1. MR Imaging

MRI is one of the most powerful in vivo imaging modalities, providing anatomical, physiological and molecular information. Using the currently available magnetic field powers, a 10–100-μm spatial resolution can be acquired in small animals. However, the low sensitivity of MRI requires the use of exogenous contrast agents, such as iron oxide or gadolinium [7]. MRI measures the relaxation processes of hydrogen protons when they precess gyroscopically with a net magnetic moment and a Larmor frequency. A resonant radio frequency causes the net magnetic precession to flip, and upon subsequent elimination of the radio frequency, the net magnetic moment gradually recovers to its original status; this is termed relaxation. Relaxation processes can be measured in terms of longitudinal (T_1_) and transverse (T_2_) relaxation. MRI contrast agents change the surrounding MRI signal intensity by shortening either the T_1_ or T_2_ relaxation time. T_1_ contrast agents produce bright MR images. T_1_ contrast agents, such as gadolinium (Gd), have been widely applied in angiography and in imaging of the gastrointestinal system, liver and whole body. A shortcoming of Gd is its toxicity, which leads to a side effect called nephrogenic systemic fibrosis [27]. T_2_ contrast agents increase the darkness of MR images and have advantages of size control with different magnetic properties, large-scale production, low toxicity, magnetic thermal effects and dragging effects caused by an external magnet [28]. Iron oxide (Fe_3_O_4_) is the most popular T_2_ contrast agent.

**Figure 2:**
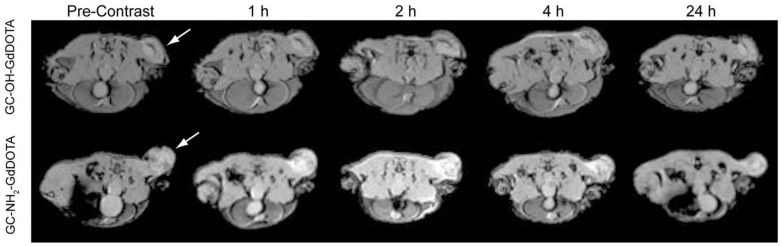
T_1_-weighted axial-view magnetic resonance (MR) images of nu/nu mice T6-17 flank tumors 1, 2, 4 and 24 h after injection of 30 μmol/kg contrast agent. Tumor areas are indicated by white arrows. Abbreviations: GC, glycol chitosan polymer; DOTA, 1,4,7,10-tetraazacyclododecane-1,4,7,10-tetraacetic acid 1-(2,5-dioxo-1-pyrrolidinyl) ester (Adapted with permission from [31]. Copyright 2013 American Chemical Society). GC, glycol chitosan.

MR contrast agents using chitosan nanoparticles have been reported for cancer MR imaging [29,30,31,32,33,34,35,36]. Nwe et al. reported T_1_ contrast agents using 1,4,7,10-tetraazacyclododecane-1,4,7,10-tetraacetic acid 1-(2,5-dioxo-1-pyrrolidinyl) ester (DOTA-NHS) and water-soluble glycol chitosan (GC) polymer, followed by Gd chelation [31]. Two hours after injection of chitosan nanoparticles into tumor-bearing mice, positive contrast effects were clearly observed in the tumor microenvironment (Figure 2). For T_2_ contrast effects, superparamagnetic iron oxide (SPIO) nanoparticles are mostly used due to their properties of low magnetic aggregation. SPIO nanoparticles have been encapsulated in the cores of hydrophobically-modified chitosan [29,33,35,36].

### 2.2. Optical Imaging

In vivo optical imaging is safe and highly sensitive and also provides fast and real-time imaging. Its major restrictions are light scattering, autofluorescence and absorption by adjacent tissues, water and lipids in the body. Recently, advanced techniques, such as fluorescence, bioluminescence, diffuse optical tomography and optical coherence tomography, have improved these limitations [37]. In particular, NIRF imaging is widely applied for in vivo small animal imaging. The NIRF imaging system utilizes the NIR window (700–900 nm) to identify the fundamental processes at the subcellular levels, minimizing the issues of autofluorescence or high absorption. NIRF imaging is possible because hemoglobin from red blood cells demonstrates major absorbance and autofluorescence in the visible region only, and water and lipids absorb primarily infrared light, not NIR [38].

**Figure 3:**
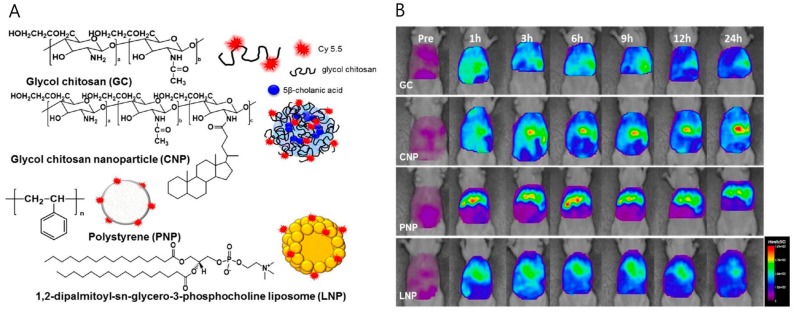
(**A**) Chemical structures of glycol chitosan (GC), glycol chitosan–5β-cholanic acid nanoparticle (CNP), polystyrene nanoparticle (PNP) and 1,2-dipalmitoyl-*sn*-glycero-3-phosphocholine liposome (LNP); (**B**) in vivo NIRF images of liver tumor-bearing mouse model at 0, 1, 3, 6, 9, 12 and 24 h after systemic injection of Cy5.5-labeled nanoparticles (Adapted with permission from [42]. Copyright 2016 American Chemical Society).

Many kinds of chitosan-based imaging nanoagents that use various NIR fluorophores have been reported. In particular, this section introduces some examples of chitosan-based imaging nanoagents that include synthetic fluorophores and synthetic fluorescence semiconductor nanocrystals. Srinivasan et al. applied IR820 fluorophore-chitosan (IR820-chitosan) conjugates, which were formulated by covalent bonding of chitosan to a carboxyl IR820, and studied them for cancer imaging. The IR820-chitosan conjugates were able to generate heat from an 808-nm laser source and, thus, can be used for photothermal therapy [39]. Zhu et al. demonstrated a CD147 antibody that was coupled with α-hederin chitosan nanoparticles, with an average particle size of 148.23 nm. The CD147 antibody mediated internalization via a caveolae-dependent pathway and lysosomal escape. The α-hederin worked by inducing apoptosis of cancer cells. For in vivo imaging, the nanoparticles included cyanine 7 (Cy7) fluorophore labels. The stronger fluorescence intensity in tumor tissues treated with α-Hed-CS-CD147-NPs indicated the higher targeting efficiency compared to that of α-Hed-CS-NPs, resulting in greater antitumor efficacy [40]. In addition to Cy7 fluorophores for in vivo NIRF imaging, many studies have used cyanine 5.5 (Cy5.5) fluorophores with chitosan polymer [41]. For example, Na et al. reported the advantages of chitosan-based nanoparticles for liver tumor imaging by comparing Cy5.5-tagged liposome nanoparticles, polystyrene nanoparticles and glycol chitosan-5β-cholanic acid nanoparticles [42]; they found that glycol chitosan nanoparticles modified with 5β-cholanic acid were sufficiently stable to maintain their structure in the bloodstream and also exhibited a prolonged blood circulation half-life of 12.2 h, longer than that of other nanoparticles studied (Figure 3). As synthetic fluorescence semiconductor nanocrystals, quantum dots have some advantages over fluorophores in terms of their bright photoluminescence, narrow emission wavelength and excellent photostability without bleaching. Quantum dots encapsulated by chitosan nanoparticles have been reported. For example, Alexandra et al. reported quantum dot cores and tripeptide-modified chitosan organic shells. These nanoparticles were designed for targeting by RGD peptides and imaging via quantum dots. The RGD peptide was conjugated with chitosan and coated onto quantum dots [43]. Although quantum dots have some advantages over fluorophores, there are still some limitations on the design of quantum dot-based nanoparticles in terms of their high reticuloendothelial system (RES) uptake and potential toxicity [44].

In vitro and in vivo optical imaging of chitosan nanoparticles has been actively reported. In the area of optical imaging, numerous types of available information and various possibilities for exploiting nanoparticles have been suggested. The only issue remaining is how to successfully translate this information by means of clinically available imaging modalities, such as MRI, CT or PET. The multimodal approaches in the next section suggest possibilities to effectively transfer current benchtop techniques to clinical use.

### 2.3. Multimodal Imaging

**Figure 4:**
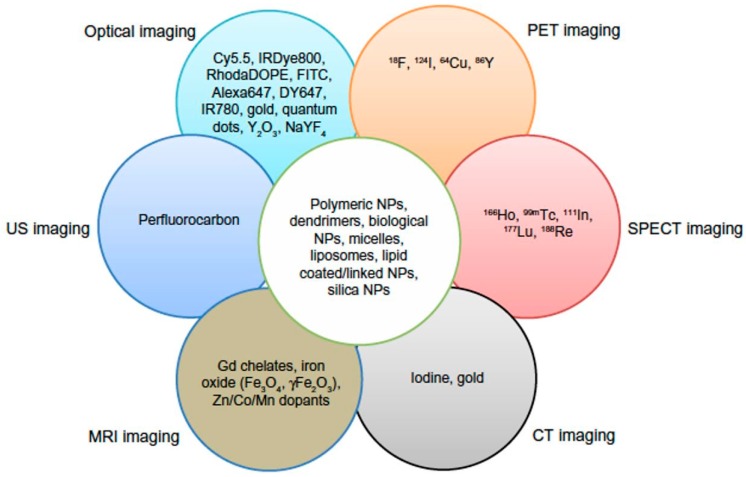
Incorporation of multicomponent imaging agents in a nanoplatform for multimodality imaging (Adapted with permission from [7]. Copyright 2014 DOVE Medical Press).

Single imaging modalities often miss small tumors due to their limited capabilities of visualization inside the body (Table 1). False negative cancer diagnostic results can shorten patient survival [1]. Thus, multimodal imaging using multimodal nanoparticles provides more information by combining benefits and compensating for the limitations of the single imaging modalities. To date, various combinations have been reported that cover dual-modal, tri-modal or other imaging modalities, such as MR-optical imaging [36], MRI-PET [45], optical imaging-CT [46], optical imaging-PET [47] and MRI-CT [48] (Figure 4) [3]. Many applications for multimodal imaging have been tested with various nanoparticles, and this section introduces some examples of MRI-optical imaging using chitosan nanoparticles.

**Figure 5:**
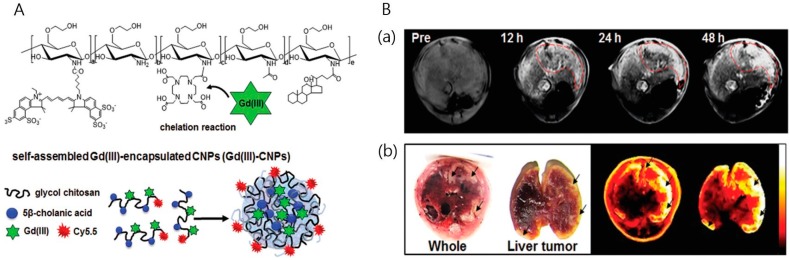
(**A**) Chemical structures of Cy5.5-labeled and DOTA-conjugated glycol chitosan-5β-cholanic acid nanoparticles; (**B**) T_1_-weighted MR and optical images of liver tumor-bearing mice after injection of Cy5.5-labeled Gd(III)-chitosan nanoparticles: (**a**) axial view of liver tumor-bearing mice pre-injection and 12, 24 and 48 h post-injection (red dots mark the tumor area, demonstrating positive contrast effects); (**b**) bright field and NIRF liver cancer images indicated by black arrows (Adapted with permission from [49]. Copyright 2016 Royal Society of Chemistry).

Neither MRI nor optical imaging use ionizing radiation, so their combination is relatively safe compared to CT or PET. The MRI-NIRF combination is interesting because it visualizes deep tissues inside the body using MRI, achieving high-resolution visualization of soft tissues and providing molecular imaging using NIRF signals. For example, MRI-NIRF multimodal imaging can be achieved by means of multiple chemically- or physically-incorporated components, such as iron oxide or Gd for MRI and NIRF imaging probes, including Cy5.5. Na et al. have demonstrated Gd(III)-encapsulated glycol chitosan nanoparticles to visualize tumors with T_1_-weighted MR imaging. They used DOTA-modified chitosan nanoparticles to chelate Gd(III), yielding particles with an average size of about 280 nm. For MR-optical multimodal imaging, they chemically conjugated Cy5.5 onto Gd(III)-chitosan nanoparticles. Both NIRF imaging and MR imaging were demonstrated in a liver tumor model (Figure 5). The chitosan nanoparticles were beneficial for liver tumor accumulation by means of EPR [49].

**Figure 6:**
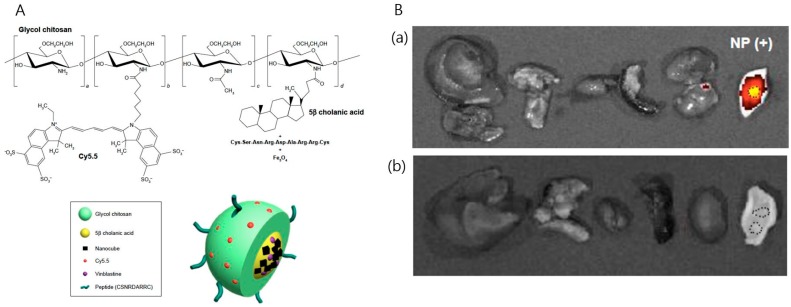
(**A**) Chemical structures and schematic diagram of glycol chitosan conjugated to hydrophobic 5β-cholanic acid and bladder cancer-targeting peptide (CSNRDARRC) and physically loaded with iron oxide nanocubes (NCs); (**B**) the combination demonstrated a specific tumor-targeting capability with minimized accumulation in other organs: (**a**) after NP administration and (**b**) before NP administration (Adapted with permission from [9]. Copyright 2016 DOVE Medical Press).

The combination of iron oxide and Cy5.5 provides a T_2_ MR contrast effect in NIRF imaging. For example, 22-nm iron oxide nanocubes, Cy5.5 and a bladder cancer-targeting peptide (CSNRDARRC) can be combined in modified glycol chitosan nanoparticles by using hydrophobic moieties. Interestingly, the resulting multicomponent system changes not only the visualization capability, but also the biodistribution of the nanoparticles in the body, changing RES uptake features in organs and enhancing tumor accumulation (Figure 6) [9].

## 3. Effective Tumor Treatment Using Chitosan Nanoparticles 

Chemotherapy uses antineoplastic drugs to treat a broad range of malignancies. A current limitation is that the drugs are also very toxic to normal cells, leading to many side effects. Minimizing side effects and maximizing therapeutic effects with reduced dosage are thus crucial objectives for improved chemotherapies.

### 3.1. Chemotherapy

Various nanoparticles have been reported for the delivery of chemotherapeutic drugs. Several nanoformulations, including liposomes, proteins and polymeric nanoparticles, are currently used in clinical applications [50]. Until now, nanoparticle delivery systems approved by the FDA have been based on liposomes, polymers or micelles. Two representative clinically-approved nanoparticle-based drugs are Doxil^®^ and Abraxane^®^. Doxil^®^ is a PEGylated liposomal doxorubicin approved in 1995 for HIV-related Kaposi’s sarcoma and metastatic breast and ovarian cancers [51]. Abraxane^®^ is paclitaxel loaded by albumin; it is used for breast cancer, non-small-cell lung cancer and pancreatic cancer. The nanoparticle delivery systems of chemotherapeutic agents suggest opportunities to more precisely target the delivery of drugs, to improve the solubility of hydrophobic drugs, to reduce immunogenicity and to increase the amount of drug in the tumor compared with the amount of drug in the heart [6,52]. In addition, nanoparticles provide controlled release of chemotherapeutic agents in subcellular compartments, such as endosomes and lysosomes, while not triggering the *p*-glycoprotein pump; this pump is known to promote multidrug resistance, acting by expelling drugs from tumor cells [53]. Although numerous therapeutic nanoparticles have been developed, the amount of untargeted nanoparticles in the body must be reduced to achieve the final goal of maximizing therapeutic effects using minimal amounts of drugs [54].

**Figure 7:**
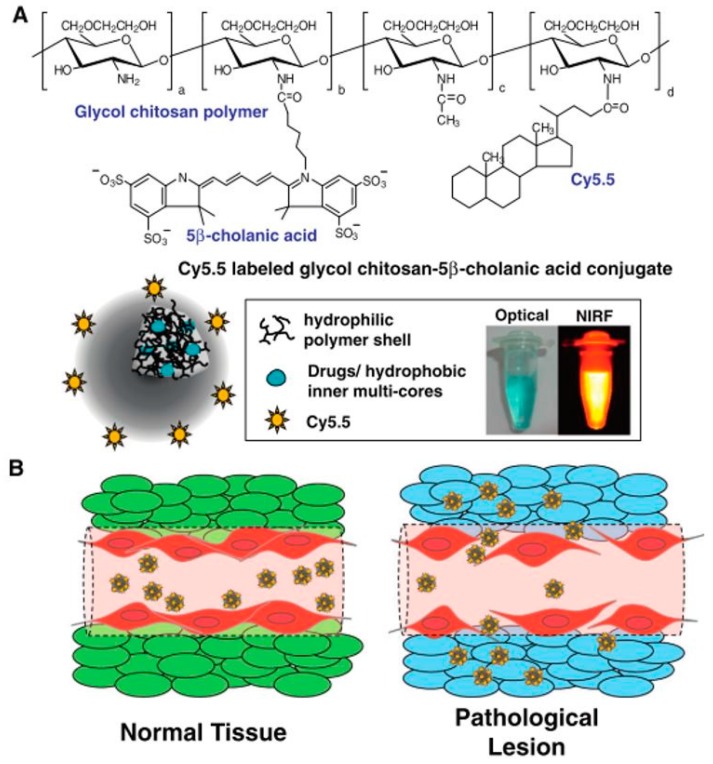
(**A**) Chemical structure and schematic diagram of hydrophobically-modified glycol chitosan nanoparticles for use in cancer diagnosis and therapy; (**B**) schematic diagram illustrating the accumulation of nanoparticles in pathological lesions (Adapted with permission from [41]. Copyright 2014 Elsevier B.V.).

Chitosan nanoparticles have been investigated as promising vehicles for the delivery of chemotherapeutic agents and cancer imaging agents [55]. Many chitosan nanoparticles have been used to deliver chemotherapeutic drugs to tumors by means of the EPR effect [20]. In particular, hydrophobically-modified glycol chitosan nanoparticles have been extensively studied (Figure 7) [41]. For example, the effect of polymer molecular weight was evaluated in terms of the tumor targeting characteristics through in vivo studies in which various amphiphilic glycol chitosan nanoparticles (GC-20 kDa-NP, GC-100 kDa-NP and GC-250 kDa-NP) were compared with respect to the hydrophobic degree of substitution, surface charge, diameter, in vitro stability and tumor accumulation [56]. Min et al. demonstrated the use of hydrophobically-modified glycol chitosan nanoparticles to deliver camptothecin (CPT); insoluble CPT was encapsulated in the glycol chitosan nanoparticles by means of a dialysis method, with loading efficiency above 80% [25]. The particles had a hydrodynamic diameter of 280–330 nm in aqueous media and showed sustained release for one week. Kim et al. also reported multifunctional glycol chitosan nanoparticles for cancer theragnosis. These nanoparticles showed distinctive characteristics in terms of stability in serum, deformability and rapid uptake by tumor cells; they were labeled with Cy5.5 for NIRF imaging and were also loaded with the anticancer drug paclitaxel [55]. More recently, Rao et al. reported chitosan-decorated nanoparticles, including encapsulated doxorubicin, for targeting and eliminating tumor-reinitiating stem-like cancer cells, which are known to cause cancer recurrence after chemotherapy. The nanoparticles specifically targeted the CD44 receptors of these cells and released the doxorubicin into the acidic tumor microenvironment. The cytotoxicity of the nanoparticles was about six-times higher than that of free doxorubicin. Finally, the nanoparticles reduced the size of tumors in an orthotopic xenograft tumor model [57].

More recent efforts using chitosan nanoparticles have used active targeting or smart targeting applications triggered by the tumor environments rather than exploiting only the EPR effect as a passive targeting method. However, it is not easy to conclude that the introduction of active or smart targeting increases tumor accumulation of the nanoparticles compared to passive targeting. Surface coverage by the targeting ligands may result in non-specific accumulation of nanoparticles in the liver or spleen via RES recognition. Nevertheless, active targeting through in vitro tests usually demonstrates higher internalization of the nanoparticles into specific cancer cells via receptor-mediated endocytosis [9].

### 3.2. Gene Therapy

With advances in genomics and proteomics, various pathways of cancer have been discovered and have led to the identification of new solutions to treat cancer at the gene level. Cancer cells are clearly differentiated from normal cells by the mutation of single or multiple genes. Thus, cancer gene therapy has emerged as a promising method to recognize cancer and treat it at the gene level. Gene therapy has two main approaches. The first is gene augmentation to upregulate tumor suppressor genes. For example, gene TP53 encodes the p53 protein and thus is known as a tumor suppressor gene [58]. The second approach involves gene knockdown by means of agents, such as short interfering RNA, siRNA [59]. For successful gene therapy using nanoparticles, the therapeutic genes have to be protected from various gene cleavage enzymes and finally transported into the targeted intracellular compartments [60].

A variety of nanoparticles have been investigated for effective in vivo siRNA delivery. siRNA agents have been encapsulated within the hydrophilic cores of lipid or polymeric nanoparticles or attached to their cationic surfaces by means of charge-charge interactions [61]. However, several studies also have indicated possible toxicity arising from the cationic properties of such nanoparticles, which can cause cell contraction, mitotic inhibition, aggregation in blood and inflammatory response [62]. Another important issue in siRNA delivery systems is related to the stability of the siRNA inside or outside the nanoparticles and within the body. The low charge density of the nanoparticles can lead to instability. siRNA loosely attached to nanoparticles can be easily released into the blood and undergo rapid degradation by nucleases before arriving at the target site, and strongly cationic polymers, such as polyethyleneimine, can be strongly cytotoxic to normal cells [59].

**Figure 8:**
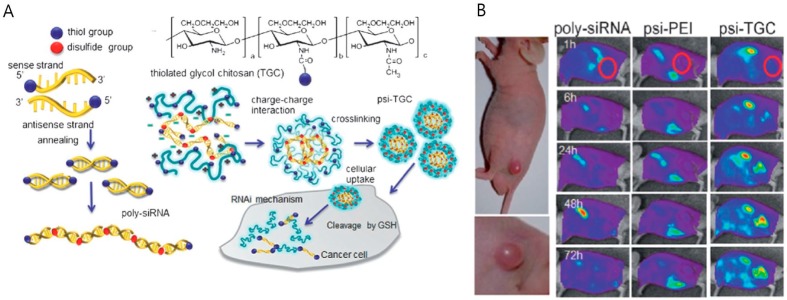
(**A**) Schematic diagram of poly-siRNA and poly-siRNA/glycol chitosan nanoparticle (psi-TGC); (**B**) whole-body images of tumor-bearing mice after injection with poly-siRNA, psi-polyethyleneimine and psi-TGC, respectively. psi-TGC showed longer circulation in the body and more accumulation at the tumor site, indicated by red circles (Adapted with permission from [59]. Copyright 2012 WILEY-VCH Veriag GmbH & Co. KGaA, Weinheim).

To achieve efficient and safe delivery of genes, alternative strategies have been suggested to increase the molecular weight of siRNAs. Polymerized siRNAs with higher molecular weights could form compounds with cationic chitosan polymers that are more stable in the body, which would enhance stability and delivery efficiency. For example, Lee et al. utilized polymerized siRNAs (poly-siRNA) and thiolated glycol chitosan polymers that were incorporated via charge-charge interaction and chemical cross-linking. The poly-siRNA glycol chitosan nanoparticles (psi-TGC) showed enhanced stability and gene silencing efficacy both in vitro and in vivo (Figure 8) [59,63].

### 3.3. Photothermal Therapy

Photothermal therapy (PTT), also known as photothermal ablation, is considered another promising method for cancer therapy. PTT utilizes various nanoscale heating agents that accumulate in tumor tissues and are then used to induce local heating that leads to cell apoptosis or necrosis depending on temperature. The local temperature is usually sufficiently above 70 °C to denature cell proteins or genes [64]. Nanoparticles work to boost the heating effects of the laser light irradiation source by means of their tuned absorption wavelengths. The areas around the nanoparticles experience effective photothermal effects, minimizing the damage to adjacent healthy tissues where only a few nanoparticles are likely to be present. Light absorption by organic dyes has been attempted for PTT, but dyes tend to lack sufficient photostability; hence, inorganic nanoparticles such as gold nanoparticles have been studied for uses in this area [65]. In particular, gold nanorods have been demonstrated because the rod shapes have uniquely strong optical absorption properties in converting NIR laser light into heat. In addition, the absorption wavelengths of gold nanorods can be tuned by means of their aspect ratio, improving their range of use as photothermal agents [66]. Chitosan polymers have been also used with gold nanorods for PTT [67,68]. Wang et al. demonstrated gold nanorods stabilized by thiolated chitosan polymer. They modified the surfaces of cetyltrimethylammonium bromide (CTAB)-passivated gold nanorods with thiolated chitosan to address the high cytotoxicity of CTAB. Additionally, the chitosan-coated gold nanorods showed better stability and biocompatibility. The chitosan-coated gold nanorods were further modified with folic acid; this increased the specificity of internalization by human colon HT-29 cancer cells, which overexpress the folate receptor. Under 808-nm NIR laser illumination, the nanorods showed potential photothermal effects [69]. Guo et al. indicated the limitations of the current PTT, which are applicable mostly at the primary site, but not to metastatic cancer. They reported NIR light–induced transformative nanoparticles that allow a combination of photothermal ablation with immunotherapy; in this approach, hollow CuS nanoparticles coated with chitosan are assembled to include immunoadjuvant oligodeoxynucleotides that contain cytosine guanine (CpG) motifs. CpG motifs act as modulators for cancer immunotherapy. The multiple components in the nanostructure are transformed after laser excitation, aiding in tumor retention of the immunotherapy. This approach was more effective than either immunotherapy or PTT alone [70].

### 3.4. Photodynamic Therapy

Photodynamic therapy (PDT) using reactive oxygen species has emerged as an important cancer therapy. PDT utilizes photosensitizers to generate cytotoxic reactive oxygen species. A specific external light wavelength is strongly absorbed by photosensitizers, producing reactive oxygen species that destroy tumors [71]. In PDT, sufficient light illumination activates photosensitizers, which destroy the malignant tumors by means of a photochemical mechanism, so the heat generated is not directly related to the therapy, unlike in PTT. The successful delivery of the illumination through the skin can induce sufficient PDT effects at the disease site. Therefore, PDT effects in internal organs can be achieved using a fiber-optic endoscopic system, whereas superficial tumors like skin cancers can be treated by direct illumination [72].

A photosensitizer activated by light produces an excited singlet state, followed by a long-lived triplet state. The triplet states undergo one of two mechanisms: in the first process, cytotoxic reactive species of free radicals and peroxides are generated by means of electron transfer with water or a biomolecule. In the second process, the triplet state reacts with oxygen and then forms a highly reactive singlet state; this second process is the most important in PDT [72]. In addition to generating singlet oxygen, photosensitizers generate a fluorescence signal. Thus, cancer-targeting nanoparticles used to deliver photosensitizers can be used for simultaneous cancer imaging and therapy. Moreover, many photosensitizers, such as porphyrins and chlorin e6 for PDT, are hydrophobic, so nanoparticles have been used to encapsulate them in their hydrophobic inner cores.

**Figure 9:**
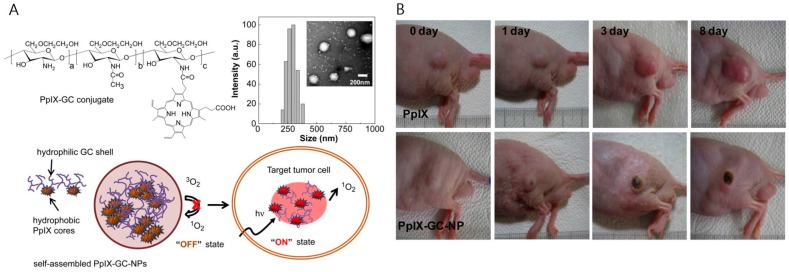
(**A**) Chemical structure and schematic diagram of protoporphyrin IX (PpIX)-conjugated glycol chitosan nanoparticles for photodynamic therapy (PDT)therapy; (**B**) result of in vivo PDT therapy by PpIX-conjugated glycol chitosan nanoparticles, compared with that of free PpIX (Adapted with permission from [76]. Copyright 2011 Elsevier Ltd.)

The use of chitosan nanoparticles for delivery of photosensitizers has been attempted [73,74,75,76,77,78]. Li et al. demonstrated photosensitizers encapsulated in chitosan-based micelles. Photosan, a porphyrin oligomer bearing sodium carboxylate groups, was applied as a photosensitizer. Photosan-loaded chitosan micelles showed higher fluorescence signals than free Photosan and generated higher levels of reactive oxygen species under laser illumination. The nanoparticles were evaluated as potential PDT agents for pancreatic cancer [73]. Lee et al. reported protoporphyrin IX (PpIX)-conjugated glycol chitosan nanoparticles for photodynamic imaging and therapy. The hydrophobic photosensitizers were chemically conjugated to glycol chitosan polymer to form a stable nanoparticle without burst release. When the nanoparticles maintained the self-assembled nanostructure, the fluorescence by the photosensitizers was weak due to the self-quenching effect. However, once the nanoparticles were inside the cells, the compact nanostructures loosened, generating strong fluorescence signals and producing singlet oxygens under laser irradiation (Figure 9) [76].

### 3.5. Hyperthermic Therapy by Magnetic Nanoparticles

Hyperthermic therapy uses iron-based magnetic nanoparticles for cancer treatment. Hyperthermic therapy uses a hyperthermic mechanism of the magnetic nanoparticles, which is induced using an alternating current (AC) magnetic field. This mechanism increases the temperature to between 43 and 46 °C, at which changes in enzymatic activities can occur, affecting cell structures and also influencing cell growth or differentiation, inducing cell apoptosis and necrosis [79].

Hyperthermia caused by magnetic nanoparticles in an AC magnetic field can be explained by two mechanisms. First, Brownian relaxation explains the heat energy of magnetic nanoparticles arising from the rotation of entire particles within their environment. Second, Neel relaxation describes the hyperthermia arising from rotations of magnetic moments inside the magnetic core [80]. The efficacy of hyperthermia is quantified by the specific absorption rate (SAR), which is defined as the rate of electromagnetic energy absorption in units of calories per kilogram or watts per gram. The absorbed energy is proportional to the temperature increase [81].

**Figure 10:**
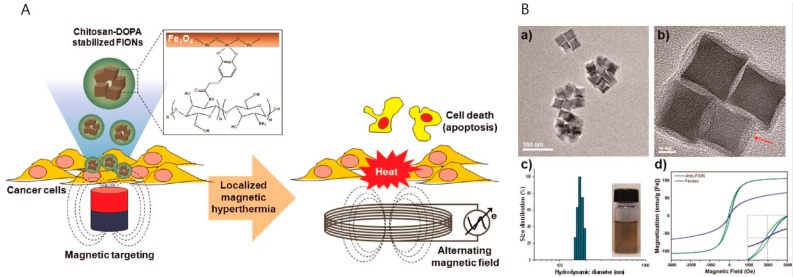
(**A**) Schematic diagram of ferrimagnetic iron oxide nanocube (FION)-loaded chitosan nanoparticles for hyperthermia of cancer cells; (**B**) TEM images and physicochemical characterizations of FION-loaded chitosan nanoparticles: (**a**,**b**) TEM images of FION-loaded chitosan nanoparticles (red arrow indicates the chitosan polymer coating layers); (**c**) hydrodynamic diameters of FION-loaded chitosan nanoparticles in phosphate-buffered saline; (**d**) magnetization curves of FION-loaded chitosan nanoparticles and Feridex^®^ (Adapted with permission from [82]. Copyright 2012 American Chemical Society).

Nanoparticles assembled from multiple smaller iron oxide nanoparticles can show higher MR contrast and more localized hyperthermic effects than freely-distributed single iron oxide nanoparticles. Chitosan polymers have been reported as a promising candidate to induce enhanced hyperthermic therapy [81,82,83]. Cervadoro et al. reported magnetic nanoparticles made by confining multiple 20-nm nanocubes into a deoxy chitosan polymer, with an overall diameter of about 156 nm. They also reported both high MR contrast effects and hyperthermic effects. Using an AC magnetic field of 512 kHz and 10 kA·m^−1^, they measured SAR values 4–15-times higher those of conventional iron oxides. Furthermore, regarding MR contrast effects, the confined nanoparticles showed a very high transverse relaxivity of about 500 mM^−1^·s^−1^ at 1.41 T [81,84]. Bae et al. reported ferrimagnetic iron oxide nanocubes (FIONs) stabilized by chitosan oligosaccharide for cancer hyperthermia. The nanoparticles were each composed of several FIONs of 30 nm in size, coated by a chitosan polymer shell. The multiple FIONs in the cores of the nanoparticles increased their total magnetic moments and locally accumulated under an applied magnetic field. The magnetic heating capability of the nanoparticle was about 31-times higher than that of commercialized superparamagnetic nanoparticles (Feridex^®^). Hyperthermic effects using these nanoparticles eliminated cancer cells through caspase-mediated apoptosis (Figure 10) [82]. 

## 4. Conclusions

Multicomponent tumor-homing chitosan nanoparticles allow unique opportunities to advance cancer therapies beyond the conventional uses of small molecules and free therapeutic agents. Thus, multicomponent chitosan nanoparticles have been extensively studied for use in various applications of multimodal imaging or theragnosis. Multimodal imaging offers the possibilities to overcome the limitations of single imaging modalities. Furthermore, the multimodal approach offers more information and supports the translation of more ideas from benchtop research to clinical use. However, because multiple components exist in these nanostructures, more unexpected factors can arise in actual clinical trials, such as additional immune responses and higher toxicities. While current successful results suggest promising future directions, many challenges remain involving the following issues: species-dependent immune responses, heterogeneous tumor vasculatures and genetic mutations with the extracellular matrix, the huge gaps between the current in vivo mouse model and actual cancer patients and patient-dependent drug efficacy. If future multicomponent chitosan nanoparticles overcome these current challenges, they will be very useful or perhaps indispensable in the future diagnosis and treatment of cancer patients.

## Figures and Tables

**Table 1:** Representative clinical and preclinical imaging modalities (Adapted with permission from [7]. Copyright 2014 DOVE Medical Press.).

Modality	Source	Typical Probes	Resolution	Depth	Sensitivity ^§^	Time Scale	Information	Cost US$	Clinical Use
MRI	Radio wave	Paramagnetic (Gd^3++^), superparamagnetic (Fe_3_O_4_)	10–100 μm	No limit	10^−9^–10^−6^	Minutes to hours	Anatomical, physiological, molecular	>$300,000	Yes
CT	X-ray	Iodine, barium sulfate, gold	50–200 μm	No limit	10^−6^	Minutes	Anatomical, physiological	$100,000–$300,000	Yes
PET	Gamma ray	Radioisotopes (e.g., ^18^F, ^11^C, ^13^N, ^15^O, ^64^Cu, ^124^I)	1–2 mm	No limit	10^−15^	Minutes to hours	Physiological, molecular	>$300,000	Yes
Optical imaging ^¶^	Light	QDs, NIRF dyes	>0.3 μm	<10 cm	10^−12^	Subseconds to minutes	Physiological, molecular	$100,000–$300,000	In development

**^§^** Moles of label detected. ^¶^ Optical imaging techniques comprise fluorescence imaging, bioluminescence imaging, fluorescence-mediated tomography and intravital microscopy. Abbreviations: MRI: magnetic resonance imaging; CT: computed tomography; PET: positron emission tomography; QDs: quantum dots; NIRF: near-infrared fluorescence.
